# Exploiting the *Crithmum maritimum* L. Aqueous Extracts and Essential Oil as Potential Preservatives in Food, Feed, Pharmaceutical and Cosmetic Industries

**DOI:** 10.3390/antiox12020252

**Published:** 2023-01-22

**Authors:** Sónia Pedreiro, Artur Figueirinha, Carlos Cavaleiro, Olga Cardoso, Maria Manuel Donato, Lígia Salgueiro, Fernando Ramos

**Affiliations:** 1 Faculty of Pharmacy, University of Coimbra, Azinhaga de Santa Comba, 3000-548 Coimbra, Portugal; 2 Associated Laboratory for Green Chemistry (LAQV) of the Network of Chemistry and Technology (REQUIMTE), University of Porto, 4099-002 Porto, Portugal; 3 Chemical Process Engineering and Forest Products Research Centre (CIEPQPF), University of Coimbra, Rua Silvio Lima, 3030-790 Coimbra, Portugal; 4 CIMAGO, Faculty of Medicine, University of Coimbra, 3001-301 Coimbra, Portugal

**Keywords:** *Crithmum maritimum* L., essential oil, chlorogenic acid, preservative

## Abstract

*Chritmum maritimum,* sea fennel, is a facultative halophyte used in salads, soups, and sauces, as well as used to prepare medicinal juices and aqueous extracts (AE) to treat several ailments. Its essential oil (EO) is used as a spice and aromatizing. In this work, the nutritional (crude protein, fiber, lipids, and ashes content) and HPLC-PDA phenolic profiles were determined. Furthermore, the antioxidant potential of the infusion and of the decoction, as well as the antibacterial activity of both, the AE and EO, were assessed against food-contaminating bacteria. The composition of the EO was also established. Sea fennel exhibited considerable fiber (34.3 ± 1.92%) and mineral content (23.6 ± 4.8%). AE contains chlorogenic acid as the major phenolic compound, 49.7 ± 0.8 mg/g in the infusion dry extract and (26.8 ± 0.9 mg/g in the decoction dry extract). EO contains high amounts of monoterpene hydrocarbons, namely γ-terpinene and sabinene. In regards to the antioxidant activity, IC_50_ values for the infusion and decoction were, respectively: 36.5 ± 1.4 μg/mL and 44.7 ± 4.4 μg/mL in the DPPH assay; 37.3 ± 2.6 μg/mL and 38.4 ± 1.8 μg/mL, in the ABTS assay. EO is particularly active against *Bacillus cereus* and *Lactobacillus plantarum*. The results support the use of sea fennel AE and EO as a potential alternative preservative ingredient for feeds, foods, pharmaceutical, and cosmetic industries, due to the antioxidant activity of infusion and decoction, and antibacterial properties of essential oil.

## 1. Introduction

Due to the exponential increase in population, food production needs to increase sustainably [1]. However, climate change and intensive agriculture are partly responsible for water scarcity, the salinization of soils, soil erosion, desertification, and the loss of biodiversity, making food production difficult [2,3,4]. To try to overcome these problems, some more sustainable approaches have emerged, including the use of halophyte plants [2,3,4]. Halophytic plants are so-called due to their ability to develop and reproduce in high-salinity environments, and can be found in coastal areas [2,3]. Furthermore, these plants are an asset from a nutritional/nutraceuticals and medicinal point of view [4,5], as they may be a healthier alternative to salt intake, a risk factor for cardiovascular [6,7] and kidney disease [8,9]. In this way, halophytic plants emerge as a value-added resource given their potential in the food, pharmaceutical, cosmetic, and veterinary industries.

*Crithmum maritimum* L. (*C. maritimum*), also called sea fennel [10,11], is a perennial and edible facultative halophyte plant that belongs to the Apiaceae family, and can be found in coastal habitats in Western Europe [2,10,11,12]. Sea fennel is traditionally consumed in salads, sauces, soups, pickled in vinegar, and as condiments, due to the good sensory traits of its essential oil [10]. Additionally, its potential as a food colorant has been reported [10]. Actually, canned sea fennel is registered by the Italian Ministry of Agriculture as a traditional agrifood product [10]. In traditional medicine, the aerial parts of this plant are prepared in the form of infusions [10], decoctions [10], and juices [10] to prevent, treat, or alleviate several pathologies such as gastrointestinal disorders [10,12,13], scurvy [10,13], cough [10], cold [10], skin problems (e.g., wounds) [11], inflammation [11,12,13], infectious conditions [12,14], and liver and urogenital diseases [10,12,13]. Additionally, sea fennel is registered as a digestive, carminative, and diuretic by the Italian Ministry of Health [14]. According to the literature, sea fennel is a source of vitamin C [10,15], fatty acids such as linoleic and linolenic acid [15], carotenoids [15], minerals including potassium, sodium, calcium, and magnesium [16], aminoacids, proteins, and polyphenols [11,15,16]. Interest in polyphenols has been increasing due to their biological properties, most of which have already been proven by scientific research. Polyphenols such as phenolic acids, flavonoids and tannins are known by their multiple biological activities such as antioxidant [17,18,19,20], antimicrobial [21,22], anti-inflammatory [17,18,23,24,25], and in general oxidative stress-related diseases [26,27,28]. Regarding its polyphenol content, sea fennel is composed mainly of hydroxycinnamic acids which caffeic acid and its derivatives are the most abundant [14,16]. Accordingly, with the literature, sea fennel’s essential oil is mainly composed of monoterpene hydrocarbons [10,12]. Additionally, due to its phytochemical composition, a few biological activities have been attributed to sea fennel, mainly antioxidant [12,16,29,30], anti-inflammatory [12], and antimicrobial activities [12,16,30].

Synthetic or natural preservatives are added to perishable foods to preserve them against spoilage caused by oxidative processes, microbial contamination, and decomposition [31]. Although, the use of synthetic preservatives is regulated by competent authorities (for example, by Food and Drug Administration (FDA)), they can contribute to the onset of diseases, or aggravate them [32]. For example, sodium benzoate is a synthetic preservative used to extend the shelf life of tomato paste for more than 1 month while preserving the quality during that time. However, in the presence of ascorbic acid, a benzene product resulting from this reaction is formed [32]. Benzene is a carcinogenic molecule and, therefore, toxic for human consumption [32]. Besides food, preservatives such as parabens, isothiasolinone, organic acids, triclosan, and chlorhexidine are used in cosmetic products [33]. In high concentrations, these preservatives are effective in inhibiting microbial growth but have toxic effects for the consumers [33] such as contact dermatitis [34]. Additionally, synthetic preservatives such as parabens are used in the pharmaceutical industry to prevent microbiological contamination [35].

There are several microorganisms responsible for food degradation and cause foodborne illnesses when consumed [36,37]. Among bacteria, *Escherichia coli* (*E. coli*), *Staphylococcus aureus* (*S. aureus*), *Bacillus cereus* (*B. cereus*), *Listeria monocytogenes* (*L. monocytogenes*), *Salmonella enterica* (*S. enterica*), and *Lactobacillus* spp. including *Lactobacillus plantarum* (*L. plantarum*) [37] are responsible for foodborne diseases, resulting in hospitalization costs and economic losses for the food industry [37]. Microorganisms can also develop in cosmetic products and drugs leading to infectious diseases [34,38]. Regarding cosmetic products, the European Regulation EC No 1223/2009 states that is not necessary sterile conditions but the safety must be secured [39]. According with the Scientific Committee on Consumer Safety (SCCP), *E. coli* and other *Enterobacteriaceae* are forbidden in all types of cosmetics and *S. aureus* must be absent in 1 mL or 1 g in children products as well in eye area and mucous membranes products [40]. For these products aerobic mesophilic organisms must have a maximum limit less than 100 Colony-Forming Units (CFU)/g or mL, and 1000 CFU/g or mL for other cosmetic products [40]. Some cosmetic products such as lipsticks can act as vectors of bacteria, namely *S. aureus*, *B. cereus*, *E. coli* and other *Enterobacteriae*, facilitating their entry in human body, causing diseases [38].

As far as we know, there are no reports about the antibacterial activity of the infusion, decoction, and essential oil of sea fennel against *Bacillus cereus* and *Lactobacillus plantarum* (food contaminants). In this work, the infusion and decoction of aerial parts from *Crithmum maritimum* L. were studied relative to their antioxidant and antibacterial potential, and phytochemically characterized by HPLC-PDA. Additionally, the antibacterial activity of essential oil was determined as well as its composition by GC-FID and GC-MS.

## 2. Materials and Methods

### 2.1. Plant Material

*Crithmum maritimum* (L.) was collected in Figueira da Foz, Coimbra, Portugal and a voucher specimen (LS7) was deposited in the herbarium of the Faculty of Pharmacy—University of Coimbra.

### 2.2. Extracts Preparation and Essential Oil Isolation

Aqueous extracts (infusion and decoction) were prepared from the aerial parts of sea fennel. Plant material was air-dried at room temperature for 1 week, milled in a knife mill (KSM 2, BRAUN, Frankfurt, Germany) and sieved with a 60 mesh sieve. For the preparation of infusion, 5 g of the dry plant was added to 500 mL of boiling water and let it stand for 30 min. For the preparation of the decoction, 5 g of the dried plant was added to 500 mL of boiling water and kept cooking for 30 min. Both the aqueous extracts were filtered under vacuum, concentrated at 40 °C in a rotavapor R-114 (Büchi^®^, Switzerland) coupled to a vacuum pump V-700 (Büchi^®^, Flawil, Switzerland) and a refrigeration circulator Minichiller (Peter Huber Kältemaschinenbau AG, Offenburg, Germany). Then the concentrated extracts were frozen, freeze-dried (FTS Systems type EZDRY, Stone Ridge, New York, NY, USA) and kept at −20 °C in the dark until use. The infusion yield was 36.27% (mg/g of dried plant material) and for the decoction 46.08% (mg/g of dried plant material). The essential oil (EO) was isolated by water distillation in a modified Clevenger apparatus as described in the European Pharmacopoeia [12] yielding 0.34% (*v*/*w*).

### 2.3. Chemical Composition

#### 2.3.1. Nutritional Profile of Sea Fennel

To achieve the proximate composition, crude protein, crude fiber, lipids, and ash content were determined following the guidelines of the Association of Official Analytical Chemists (AOAC) [41]. To determine the moisture content, fresh samples of sea fennel were dried in a U-10 oven (Memmert, Schwabach, Germany) at the temperature of 105 ± 5 °C until constant weight (AOAC 934.01).

The crude protein amount was evaluated by the Kjeldahl method (N × 6.25). For that, the sample was submitted to a digestion process with concentrated H_2_SO_4_ in a compact digestion system (MBC-6/N Kjeldahl from Raypa, Barcelona, Spain) followed by distillation (Kjeldahl UDK 127 semi-automatic distillation unit from Velp Scientifica, Usmate, Italy) of the digestion product with concentrated NaOH into H_2_SO_4_ 0.2 N. Posteriorly, the sample was titrated with NaOH 0.2 N (AOAC, 2000.11). To determine the crude fiber, the Weende method was performed using a Fibertest F-6 fiber extraction system (Raypa, Barcelona, Spain). It was added to the sample H_2_SO_4_ 0.26 N and NaOH 0.32 N at 100 °C for 30 min (AOAC 962.09). Relatively to lipids content, the fats were extracted with petroleum ether at 40–60 °C in a Soxhlet apparatus being posteriorly quantified by gravimetry (AOAC 991.36). After incineration in a muffle furnace (Gallenkamp, Loughborough, England) at 550 °C ± 10 °C for 7 h, ashes content was determined by gravimetry (AOAC 930.05). Moisture was expressed on a fresh weight basis and the other determined parameters in dry matter. All determinations were performed in triplicate.

#### 2.3.2. Phytochemical Characterization

##### Phenolic Profile and Quantification of Chlorogenic Acid by High Pressure Chromatography Coupled to Photo Diode Array Detector (HPLC-PDA)

The phenolic profile of aqueous extracts of sea fennel was achieved by HPLC-PDA on a liquid chromatograph from Gilson Electronics SA (Villiers le Bel, France) with a Spherisorb S5 ODS-2 column (250 × 4.6 mm i.d., 5 µm) (Waters Milford, MA, USA) with a C18 guard cartridge (30 × 4 mm i.d., 5 µm) from Nucleosil (Macherey-Nagel, Düren, Germany), at 24 °C. The separation was performed using a mobile phase composed of formic acid 5% (*v*/*v*) and methanol with a continuous gradient of 5 to 50% of formic acid 5% for 50 min, at a flow rate of 1 mL/minute. The volume of the sample injected was 100 μL and the UV spectra acquisition was carried out between 200–600 nm. Chromatographic profiles were recorded at 280 and 320 nm. Data treatment was performed with Unipoint^®^ (version 2.10 software) from Gilson (Middleton, WI, USA). For the quantification of chlorogenic acid, several dilutions (1 to 200 µg/mL) of a chlorogenic acid standard from SIGMA (St. Lois, MO, USA) dissolved in water were prepared and analysed in HPLC-PDA as an external standard, and the absorbance recorded at 320 nm. It was performed three independent injections (100 µL) in duplicate for each sample. To evaluate the correlation between peak area and concentration, the least-squares regression model was used. The detection and quantification limits (LOD and LOQ, respectively) were determined from the calibration curve. Therefore, the quantification of chlorogenic acid was calculated from the standard calibration curve and the peak area of the compound.

##### Analysis of the Essential Oil

The composition of the oil was achieved by combination of Gas-Chromatography (GC) and Gas-Chromatography coupled Mass Spectrometry (GC-MS) analysis. GC was performed in a Hewlett-Packard 6890 (Agilent Technologies, Palo Alto, CA, USA) gas chromatograph, with a single injector and two flame ionization detectors. A divider (Agilent Technologies, part no. 5021-7148) was used for simultaneous sampling into two different fused silica capillary columns: SPB-1 (polydimethylsiloxane 30 m × 0.20 mm i.d., film thickness 0.20 μm) and SupelcoWax-10 (polyethyleneglycol 30 m × 0.20 mm i.d., film thickness 0.20 μm) (both from Supelco, Bellefonte, PA, USA). An HP GC ChemStation Rev. A.05.04 data system was used for operation and data handling. Oven temperature program: 70–220 °C (3 °C·min^−1^), 220 °C (15 min); injector temperature: 250 °C; carrier gas: helium, adjusted to a linear velocity of 30 cm.s^−1^; detectors temperature: 250 °C. GC-MS analysis was performed with an Agilent 6890 gas chromatograph interfaced with a mass selective detector MSD 5973 (Agilent Technologies), both operated by HP Enhanced ChemStation software, version A.03.00. A HP1 fused silica column (polydimethylsiloxane 30 m × 0.25 mm i.d., film thickness 0.25 μm) was used. GC parameters were as above described; MSD parameters: interface temperature: 250 °C; MS source temperature: 230 °C; MS quadrupole temperature: 150 °C; ionization energy: 70 eV; ionization current: 60 μA; scan range: 35–350 units; scans.s-1: 4.51. The oil sample was diluted (1:8) in n-pentane and then injected (0.2 μL) in split mode (1:40). Identifications of the components were achieved by considering, concurrently: the acquired retention indices on both SPB-1 and SupelcoWax-10 columns determined by linear interpolation relative to the retention times of C_8_–C_23_ of n-alkanes and compared with reference data from authentic products (available in the laboratory database of the Faculty of Pharmacy, University of Coimbra) and literature data [42,43] the acquired mass spectra compared with reference data from the Wiley/NIST library [44]. The relative amount of each component was determined by normalization of the peak area without any correction.

### 2.4. Evaluation of Biological Activities

#### 2.4.1. Antioxidant Activity

The antioxidant activity of the aqueous extracts from sea fennel (infusion (INF) and decoction (DEC)) were evaluated in DPPH and ABTS assays.

##### 2,2-Diphenyl-1-Picrylhydrazyl Radical Assay (DPPH)

The DPPH method was used to assess the free radical-scavenging activity of sea fennel infusion and decoction, as described in Pedreiro et al., (2021) [17]. Aliquots of 100 µL were assayed in 500 µL of a methanol 500 µM solution and 100 µL of acetate buffer 100 mM (pH 6.0). The reaction mixtures (300 µL) were incubated for 30 min at room temperature, and under light. After 30 min, the absorbance was measured at 517 nm in a Thermo scientific multiskan FC plate reader. The IC_50_ (minimum concentration that reduces DPPH by 50%) was calculated and the results were expressed as IC_50_. For comparison, the free radical-scavenging activity of butylated hydroxyanisole (BHA) was assessed. All the determinations were performed in triplicate.

##### 2,2′-Azinobis-(3-ethylbenzothiazoline-6-sulfonate) Assay (pH = 7) (ABTS)

The ABTS radical was generated by the oxidation of ABTS^●+^ 7 mM with potassium persulphate 2.45 mM (Merck, Darmstadt, Germany) in water, and stored for 12 to 16 h under the light, at room temperature [45]. Posteriorly, buffered saline (PBS) at pH 7.0 was added to this solution to achieve an absorbance of 0.7 ± 0.02 at the wavelength of 734 nm. Then, 50 µL of the sample was mixed with 2 mL of ABTS^●+^ and vortexed for 10 min. The reaction was incubated for 4 min being its absorbance measured at 734 nm. The correspondent IC_50_ value was calculated. All the determinations were performed in triplicate.

#### 2.4.2. Antibacterial Activity

The antibacterial activity of aqueous and essential oil extracts from sea fennel was assessed using the agar diffusion method and microdilution assay to achieve the minimum inhibitory concentration (MIC) and minimum bactericidal concentration (MBC). The antibacterial activity was evaluated in *Escherichia coli* ATCC 8739 (*E. coli*), *Staphylococcus aureus* ATCC 29213 (*S. aureus*), *Bacillus cereus* isolated from UCU [46], and *Lactobacillus plantarum* DSMZ 20205.

##### Disc-Diffusion Assay

In disc-diffusion method, Mueller-Hinton agar (VWR Chemicals, Radnor, PA, USA) was prepared and placed in Petri dishes. For *E. coli* and *S. aureus* inoculum of 1.5 × 10^8^ CFU (0.5 McFarland standard, BioMérieux SA, Marcy-l’Etoile, France) of bacteria/mL was used for completed seeding of the agar, and inoculum of 0.75 × 10^8^ and 3 × 10^8^ CFU of bacteria/mL was used for *B. cereus* and *L. plantarum*, respectively. Filter paper discs of 6.0 mm in diameter (Liofilchem, Roseto degli Abruzzi, Italy) were impregnated with several concentrations of infusion, decoction and essential oil, placed in the seeded agar and incubated at 37 °C for 24 h. For solvent control, DMSO 15% (solvent used for essential oil dissolution) was used. The diameter of the inhibition zone around the filter paper disc was measured in millimetres. All the determinations were performed in triplicate, and the results were expressed as mean ± standard deviation (SD).

##### Microdilution Assay

Minimum inhibitory concentrations (MICs) and minimum bactericidal concentrations (MBCs) of essential oil were determined by microdilution assay (https://www.eucast.org/ast_of_bacteria/mic_determination, accessed on 30 May 2022). The MICs and MBCs of aqueous extracts were not determined due to the lack of activity observed in the disc diffusion method. The bacterial suspensions of *E. coli* and *S. aureus* were prepared with turbidity of 0.5 McFarland, and for *B. cereus*, and *L. plantarum* of 0.25 and 3 McFarland, respectively and 1.5 μL were added to each well. The plates were incubated for 24 h, at 37 °C and the absorbance was measured at 620 nm in a Thermo scientific multiskan FC plate reader, at 620 nm. For MBCs determination, 10 μL of the wells where growth inhibition was observed, was pipetted to Mueller-Hinton agar and incubated at 37 °C for 24 h, being colonies (if any) counted. MBCs were achieved when 99.9% of the final inoculum was killed after 24 h as described in Balouiri et al., (2016) [47]. For solvent control, DMSO 7.5% was used, to verify if the solvent affects bacterial growth. Additionally, negative and positive controls were performed. This procedure was repeated for all bacterial strains and negative and positive controls. All the experiments were performed in triplicate, and the results were expressed as mean ± SD.

### 2.5. Statistical Analysis

Statistical analysis was carried out by GraphPad Prism version 5.01 (GraphPad Software, San Diego, CA, USA). Paired t-test was performed to compare the IC_50_ values of infusion and decoction, in the ABTS assay. For statistical analysis of the IC_50_ values of infusion, decoction and BHA obtained in DPPH assay, a One-Way ANOVA followed by Tukey’s test was performed. The level of significance for both statistical tests was set at *p* < 0.05.

## 3. Results

### 3.1. Nutritional Profile of Sea Fennel

Accordingly to Table 1, sea fennel has low water content and a significant amount of crude fiber, and ashes. Relatively to moisture, the obtained result is corroborated by literature [10]. Plants/foods with low moisture amounts have a higher shelf life due to their slow deterioration [48]. Sea fennel presents a low quantity of protein although a little higher than reported by Martins-Noguerol et al., (2022) [49]. Relatively to lipids, the result is in concordance with the literature [49]. However, the nutritional content, particularly protein and lipids, can be improved in optimal growth conditions of sea fennel [49]. The mineral content is a significant amount which is corroborate by the mineral content reported, particularly Na, K and Fe [10,50].

### 3.2. Phenolic Profile of Aqueous Extracts from Sea Fennel

**Figure 1:**
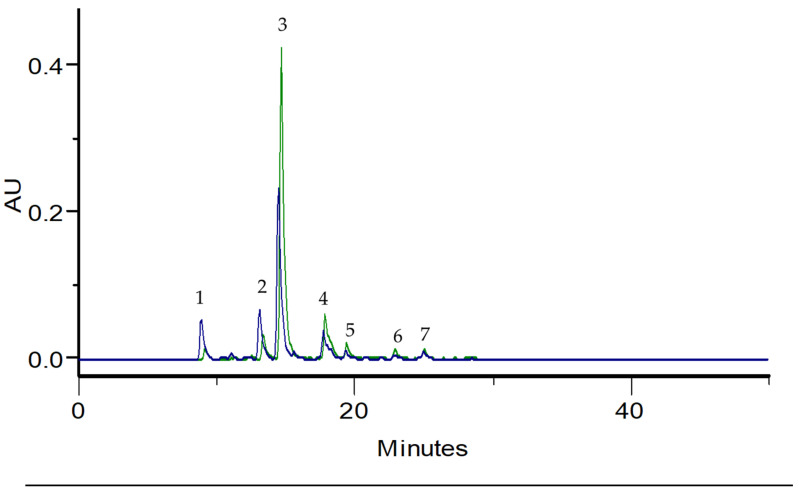
HPLC-PDA profiles of *Crithmum maritimum* L. infusion (green line) and decoction (blue line), recorded at 320 nm. Peaks 1–3 and 5–7 are caffeic or ferulic acid derivatives. Peak 4 is a *p*-coumaric acid derivative.

According to Figure 1, and the respective absorption spectra, both samples have a similar composition, being constituted mainly by phenolic acids. Peaks 1 to 3 and 5 to 7 correspond to caffeic or ferulic acid derivatives due to their UV absorption spectra profile (maximum absorption of 326 nm and a shoulder at 298 nm) [17,51]. In the UV absorption spectra, peak 4 presented a shoulder at 290 nm and a UV absorption maximum at 312 nm indicating the presence of a *p*-coumaric acid derivative [51]. Although both chromatograms present similar profiles and composition, it is evident that, in the case of the sea fennel’s decoction (Figure 1) the area of peak 3 is lower and peaks 1 and 2 areas higher than the correspondent peaks area in the infusion chromatogram.

There are a few reports about the phytochemical characterization of sea fennel extracts, in which phenolic acids were identified as the main molecules, particularly quinic acid derivatives [10,11,29,52,53]. Within these, chlorogenic acid, an ester of caffeic and quinic acids, was identified as the major compound [10,11,52,53,54]. In another study, the phytochemical characterization of infusion and decoction of sea fennel was performed by HPLC-PDA [29]. The results showed that both aqueous extracts were composed mainly of phenolic acids being the chlorogenic acid the major compound [29], which corroborates our results. Our group characterized the hydrodistillation residual water (HRW) of sea fennel by HPLC-PDA-MSn [12]. The chromatographic profile of HRW was identical to the infusion and decoction HPLC-PDA chromatograms. The authors identified phenolic acids as the main components of HRW, being chlorogenic acid (5-caffeoylquinic acid) the major compound. However, although in minor content, flavonoids namely apigenin and quercetin glycosides were detected [12], which is not in accordance with our results, probably due to the extraction nature (extraction occurred during 3 h, at 100 °C). Thus, in an attempt to confirm the chlorogenic acid composition of the samples, the chlorogenic acid standard was analysed by HPLC-PDA, being the retention time of the peak identical to the chromatogram’s aqueous extracts of sea fennel. Therefore, the chlorogenic acid was quantified in both aqueous extracts, by HPLC-PDA.

#### Quantification of Chlorogenic Acid

The equation of the calibration curve of chlorogenic acid was y = 3,672,048.16ϰ + 5,519,862.44 being the linear correlation coefficient (r^2^) 0.9998. The mean of the areas of infusion and decoction were interpolated in the calibration curve of chlorogenic acid. Thus, the concentration of chlorogenic acid in infusion and decoction were 49.7 ± 0.8 and 26.8 ± 0.9 mg/g of extract, respectively. LOD and LOQ were 1.1 ± 1.0 and 7.2 ± 0.9 mg/g of extract, respectively. Our results are in line with the area of peak 3 (correspondent to chlorogenic acid) of chromatograms of infusion and decoction (Figure 1) which is higher in the former than the latter. This difference can be due to the extraction conditions [55]. In decoction, the powdered material of sea fennel was in boiling water at 100 °C for 30 min while in the infusion the temperature of the water was decreasing during the 30 min of extraction. Since polyphenols are thermolabile, in the former due to high temperatures, degradation of polyphenols, mainly chlorogenic acid derivatives (major compound) could happen [55]. Alemán et al., (2019) studied an aqueous and ethanolic extract of sea fennel and quantified the chlorogenic acid in both extracts [53]. The amount of chlorogenic acid in the aqueous extract was 42.6 mg/g and for ethanolic extract 58.5 mg/g of plant (dry weight) [53]. In our results, the chlorogenic acid in sea fennel’s infusion is slightly higher, probably due to the type of extraction [55,56]. Note that the aqueous extract that Alemán et al., (2019) studied is not an infusion, being the extraction conditions different which influence the phytochemical composition as well as the amounts of compounds extracted [55,56]. In another study, infusion and decoction of sea fennels were prepared and its content of chlorogenic acid was studied [29]. The results displayed by the authors showed an identical content of chlorogenic acid in both aqueous extracts which are not in line with our results. This fact may be due to the extraction time since the extraction time was 5 min, both in the infusion and in the decoction. Once again, the extraction time of our extracts was 30 min and, therefore, there may have been thermal degradation of the phytoconstituents.

### 3.3. Sea Fennel’s Essential Oil Composition

Table 2 summarizes sea fennel’s essential oil composition. The EO is mainly composed by monoterpenic hydrocarbons (74.4%) followed by oxygen-containing monoterpenes (24.8%). γ-Terpinene (37.2%) is the major constituent followed by sabinene (21.2%). Thymylmethyl oxide (16.4%) is the most representative oxygen containing monoterpene. The composition of the EO is in accordance with results previously described in the literature [12,57], only with slight quantitative differences in the relative amounts of monoterpenic hydrocarbons and oxygen-containing monoterpenes. However, other chemical profiles have been also identified in *C. maritimum* from different origins. Indeed, EO from plants obtained in Croatia, Turkey, and Italy [52,57,58,59] have been reported as being rich in limonene; β-phellandrene has also been reported in high amounts in the essential oil from *C. maritimum* collected in Turkey [60]. Dillapiole was also detected in significant amount [60].

The achieved differences can be explained due the different edaphoclimatic conditions of the geographical origins of the samples that may affect the oils compositions [61] as well by the existence of different chemotypes as is suggested by Ozkan et al., (2001) [57].

### 3.4. Antioxidant Activity

The antioxidant activity of aqueous extracts from sea fennel was evaluated by DPPH and ABTS methods (Table 3). According to Table 3, the infusion is more active relatively to the decoction. This difference can be attributed to the higher content of phenolic acids, particularly in chlorogenic acid, in infusion as observed in Figure 1. Additionally, in DPPH, BHA is approximately 10 times more active relatively to the aqueous extracts of sea fennel. Siracusa et al., (2011) studied the infusion of sea fennel, collected in the flowering station in Croatia [62]. The results showed a DPPH inhibition of 88% at 400 mg/mL being this activity attributed to the chlorogenic acid, identified as the major phenolic acid [62]. Although the result is not expressed as an IC_50_ it is clear that the free radical-scavenging activity of our extracts (e.g., infusion and decoction) has higher activity. Souid et al., (2021) studied the antioxidant activity by DPPH of an hydro-ethanolic extract from sea fennel [30], obtaining an IC_50_ value of 0.22 ± 0.04 mg/mL, being significantly less active than our samples. The hydroethanolic extract was constituted by phenolic acids and, in minor amounts, by flavones and flavonols. Similar to our results, chlorogenic acid was the main compound. However, its amount in the hydro-ethanolic extract was 7.25 mg/g (dry weight) [30], which is significantly lower than the chlorogenic acid content in our aqueous extracts. Comparing the results obtained by Souid et al., (2021), it is evident that antioxidant activity observed for aqueous and hydro-alcoholic extracts is related to the type of solvent and extraction conditions that influences the phenolic compounds composition and its quantity. Relatively to the BHA antioxidant activity, Durmaz et al., (2022) reported an IC_50_ value of 10.10 μg/mL [63].

### 3.5. Antibacterial Activity

The antibacterial activity of sea fennel’s aqueous extracts and essential oil was evaluated against *B. cereus*, *S. aureus*, and *L. plantarum* (gram-positive bacteria) and *E. coli* (gram-negative bacteria). The results of the antibacterial activity of sea fennel’s EO in disc diffusion assay and MICs and MBCs values are presented in Table 4 and Table 5, respectively. Accordingly with the inhibition halo presented in Table 4, the EO exhibited higher antibacterial effect in *L. plantarum*, followed by *B. cereus*. The MICs values of EO (Table 5) were the same for *B. cereus* and *L. plantarum* (11.4 ± 0.1 μL/mL) which presented the highest activity followed by *S. aureus* and *E. coli*. At the tested concentrations, the infusion and decoction of sea fennel did not exhibit any antibacterial effect.

## 4. Discussion

The infusion and decoction of sea fennel showed a significant free-radical scavenging activity in both DPPH and ABTS assays, being approximately 10 times less active than BHA. This synthetic preservative it is widely used in food industry, particularly in oils and foods rich in fats, to increase their shelf-life [64]. Although BHA is considered and generally recognized as safe (GRAS), this molecule has been reported to have negative effects in health [64], namely in carcinogenesis [65]. In high doses, BHA induced cancer of forestomach in rats [64]. Additionally, this synthetic preservative, when irradiated with visible light, and in the presence of vitamin B2, suffers degradation [64]. Thus, the FDA established that the use of BHA must be lower than 0.02%, alone or in combination with other preservatives [64]. Additionally, the Joint Food and Agriculture Organization of the United Nation/World Health Organization Expert Committee on Food Additives (JECFA) determined that the daily intake of BHA was 0.5 mg/kg [64]. However, a study performed in the Netherland with 120,852 older adults, determined that the daily intake of BHA was 105 μg/day [64], a value higher than the recommended. In this way, the addition of natural preservatives prevents the negative effects in health related to high intake of synthetic preservatives such as BHA. Additionally, the addition of plant extracts to food matrices improves the nutritional features of the food. Sea fennel emerge has healthier antioxidant product, being environmentally friendly and economic due to its growth and development characteristics. Regarding the phytochemical characterization, both extracts revealed to have similar compositions in which chlorogenic acid was identified as the major compound. Polyphenols, including phenolic acids, are known for their ability to neutralize free radicals. Thus, the antiradical activity exhibited by aqueous extracts can be attributed to their phenolic composition, namely chlorogenic acid.

Chlorogenic acid has been reported as an antioxidant molecule, due to its structure which contains 5 hydroxyl (OH) groups and one carboxylic (-COOH) group. The latter is a strong electron-withdrawing being the former electron donors [66]. Additionally, the number and position of OH groups influence the free radical neutralization capacity. Accordingly with Chen et al., (2020), if the number of OH groups in the benzene ring is less than four, the antioxidant activity is proportional to the number of hydroxyl groups [66]. Additionally, OH groups can enhance the antioxidant capacity of other polyphenols [66]. At the cellular level, chlorogenic acid was found to reduce the expression of a regulator of cellular oxidative stress response activating signaling pathways involved in the expression of endogenous antioxidant enzymes such as superoxide dismutase (SOD), catalase (CAT), glutathione peroxidase (GSH-Px), and glutathione (GSH) [67]. Chlorogenic acid present in coffee exhibited antioxidant activity in DPPH, superoxide anions, OH radicals, and peroxynitrite. However, depending on the concentration of chlorogenic acid and the presence of free transition metal ions and their redox state, this phenolic acid can act as a pro-oxidant [54]. Antimicrobial activity was also attributed to chlorogenic acid rich extracts. Due to its antioxidant activity, chlorogenic acid has potential in food industry as a preservative [54]. Lipid peroxidation are oxidative processes that occurs in foods leading to their deterioration [68]. The incorporation of phenolic compounds such as chlorogenic acid in food matrices can delay oxidative processes, increasing the product shelf life [68]. However, chlorogenic acid is sensitive to pH, high temperatures, and light exposure, which influences its biological properties, namely antioxidant activity [54,67]. Besides food applications, chlorogenic acid was found to have the potential in protecting skin photoaging triggered by UVA-irradiation through collagen degradation inhibition and enhancement of collagen synthesis [69]. In another study, the skin anticancer potential of chlorogenic acid nanoparticles was studied [70]. The chlorogenic acid nanoparticles were administrated orally and topically in mice with skin cancer induced by 7,12-dimethylbenz(a)anthracene (DMBA). The results showed that chlorogenic acid nanoparticles were capable to inhibit skin cancer cell growth and restore the levels of enzymatic and non-enzymatic antioxidants [70]. Chlorogenic acid in its free form was also tested and, although have shown to inhibit skin cancer growth, its activity was lower compared to the nanoparticles [70]. This compound is also reported to have antimicrobial activity [71,72,73,74]. However, the sea fennel’s infusion and decoction did not reveal antibacterial activity, at the tested concentrations. This fact, as mentioned earlier, may be due to the instability of chlorogenic acid in the extraction procedures [54,67].

*E. coli*, Gram-negative bacteria, is a facultative anaerobe that is present in gut microbiota of animals and humans. However, the ingestion of pathogenic strains of *E. coli* present in contaminated foods, depending on the bacteria serotype and immune system of the host, can cause diarrhoea, vomiting, nausea and infections in the urinary and central nervous system as well in the bloodstream [37]. According to the Food and Drug Administration (FDA), this coliform bacterium can be found in meats and food crops (https://www.fda.gov/food/foodborne-pathogens/escherichia-coli-e-coli). Similar to *E. coli*, the Gram-positive bacteria *S. aureus* cause identical symptoms and also toxic shock syndrome, scarlet fever and respiratory diseases [37]. Although its presence in cosmetic products is forbidden, when contaminated, *S. aureus* can cause conjunctivitis and impetigo [38]. *E. coli* and *S. aureus* can be found in mascaras, eyeliners, face powders, and lipsticks, increasing the probability of external and internal infections [38]. The saprophytic endospore-forming Gram-positive bacteria, *B. cereus* occurs naturally in a wide variety of raw materials for consumer goods [75], and is responsible for gastrointestinal intoxications [37,75,76], being present, for example, in cosmetics, and dairy products such as milk and cheese [77]. *B. cereus*, due to its capability of forming spores, can be found in many foods such as dairy products, raw meat, pasta and other processed foods [76], and also in cosmetic raw materials and finished products [75]. *B. cereus* contaminations in cosmetic products are common and, in damaged skin (e.g., wounds), can cause cutaneous infections [75]. Additionally, eye contaminated-cosmetics can cause severe eye infections [78]. *L. plantarum*, a Gram-positive aerotolerant bacteria, is commonly found in meat, fermented and processed foods that in high levels causes food spoilage [37]. Unlike the bacteria mentioned above, *L. plantarum* has antimicrobial potential against others food poisoning bacteria [79]. However, high levels of *L. plantarum*, particularly found in fats, leads to the spoilage of fats-enriched foods [37]. In cosmetics, *L. plantarum* exerts a protective effect on the skin through suppression of several harmful bacteria found in skin, and it has been reported that the absence of this lactic bacteria is associated with skin aging [80].

The essential oil of sea fennel showed antibacterial activity against *E. coli*, *S. aureus*, *B. cereus*, and *L. plantarum*, with higher activity in the last two. This activity is probably due to the EO composition, particularly to the contribution of γ-terpinene and sabinene.

Sea fennel’s essential oil has a few reports about its antibacterial activity [81,82] attributed to the high content of monoterpenic hydrocarbons. Sanchèz-Hernandèz et al., (2021) reported the antibacterial activity against *E. coli* and *S. aureus* of EO (100 μL/mL) from sea fennel [81]. The EO rich in γ-terpinene, thymol methyl ether, and dillapiole showed an inhibition halo of 13 mm in *S. aureus* and no inhibition of *E. coli* [81]. D’Agostino et al., (2021) studied the antibacterial activity of sea fennel’s EO against Bacillus sp. [82]. The EO, composed mainly by *β*-myrcene, *p*-cymene, and thymol acetate showed an inhibition halo of 10 mm in *Bacillus* sp. [82]. Another study, reports MICs values above 50 μL/mL of γ-terpinene for *Escherichia coli* and *Staphylococcus aureus* [83]. For *E. coli* and *S. aureus*, γ-terpinene inhibited the bacterial growth at concentrations above 50 µL/mL, which is in accordance with our results for *E. coli*. However, our EO from the sea proved to be more effective in inhibiting *S. aureus* growth than γ-terpinene alone, suggesting synergistic interactions. Other authors reported MICs values of γ-terpinene and sabinene higher than 10 µL/mL, in *E. coli* and *S. aureus*. Another study of the antibacterial activity of the main compounds of the EO (γ-terpinene, and sabinene) was screened against *Bacillus cereus*, *Salmonella typhimurium*, *Escherichia coli*, and *Staphylococcus aureus* [84]. These three compounds did not show activity in all the tested bacteria.

Besides its antibacterial activity reported above, Alves-Silva et al., (2020) reported the antifungal activity of γ-terpinene, sabinene, and EO of sea fennel against *Aspergillus* strains [12], a food poisoning bacteria that produces highly toxic aflatoxines [85]. The information gathered in the literature, suggest that the antibacterial activity depends on the chemical profile of the EO.

Given the antioxidant properties of aqueous extracts and antibacterial activity of essential oil, sea fennel has potential to be a future food and cosmetic preservative and, thus, replacing or decrease synthetic preservatives. *Crithmum maritimum* L. could also be used in feed industry, considering EFSA opinions based on other plants that exhibit in there composition chlorogenic acid [86] or essential oils [87] Nutritional analysis of *C. maritimum* revealed higher fiber content. Recent changes in animal production have been based on feed regulations moving away from the use of Growth Promoting Antibiotics (AGP), and therefore there is a need to find new and safe approaches to optimize the intestinal health of animals [88]. In addition to its added nutritional value, dietary fiber has been found to improve gut health and immune function in monogastric animals [88]. Due to its antimicrobial properties and organoleptic characteristics, some of the essential oils have potential to enhance the dermocosmetics properties of the finished products as well to preserve the cosmetic formulation [89]. Besides antimicrobial activity, esential oils present other functions such as anti-aging, sun protection, and skin lightning [89,90]. Additionally, essential oils contributes to hair nutrition, and moisturizer [90]. Good sensory traits has been reported for *C. maritimum* [10]. Thus, allied to antibacterial essential oil properties and antioxidant activity, *C. maritimum* have potential as a cosmetic/cosmeceutical product. Given the nutritional profile of *C. maritimum* and antioxidant activity of aqueous extracts, this plant has potential as a dietary supplement in order to prevent oxidative-related diseases [91]. Additionally, being a salt-tolerant plant with significant fiber and minerals content, sea fennel can be a future sustainable agroalimentar product contributing for the reuse of degraded soils (e.g., salinization, erosion).

## 5. Conclusions

The incorporation of *Crithmum maritimum* L. in foods and cosmetics can be an alternative to synthetic preservatives. However, more studies are needed to evaluate the effect of the incorporation of these extracts on the organoleptic characteristics of food and fodder. Due to its high content in fiber and chlorogenic acid, *C. maritimum* extracts can improve the nutritional composition of foods and feeds, and also present value as a dietary supplement. As an agroalimentar product, it can offer a sustainable solution relative to soil salinization and erosion as it is a halophyte plant, thus, a salt-tolerant crop.

## Figures and Tables

**Table 1:** Moisture (% fresh weight) and nutrient composition (% dry weight) of *Crithmum maritimum* L. (May). All the determinations were performed in triplicate. Results are expressed as mean ± SD.

Composition	Content
Moisture	14.3 ± 0.2%
Crude Protein	8.0 ± 0.1%
Crude Fiber	34.3 ± 1.92%
Lipids	5.8 ± 0.1%
Ash	23.6 ± 4.8%

**Table 2:** Composition of essential oil from *Crithmum maritimum* L.

Exp. RI^a^	Ref. RI^a^	Exp. RI^b^	Ref. RI^b^	Compound *	Percent in Sample (%)
922	922	1030	1028	α-Thujene	0.3
930	930	1030	1030	α-Pinene	1.8
943	943	1075	1073	Camphene	t
964	964	1124	1126	Sabinene	21.2
970	970	1118	1116	β-Pinene	0.3
980	980	1161	1162	Myrcene	1.4
982	983	1294	1291	Octanal	t
997	997	1171	1168	α-Phellandrene	0.8
1005	1005	1152	1152	Δ3-Carene	t
1010	1110	1187	1189	α-Terpinene	1.6
1011	1011	1275	1273	*p*-Cymene	4.2
1020	1020	1213	1214	β-Phellandrene	0.5
1025	1025	1235	1235	*Z*-Ocimene	4.2
1035	1035	1250	*1253*	*E*-Ocimene	0.2
1046	1046	1249	1251	γ-Terpinene	37.2
1050	1050	1459	1462	*E*-Sabinene hydrate	0.4
1076	1078	1288	1288	α-Terpinolene	0.6
1080	1081	1444	1445	*Z*-Sabinene hydrate	0.2
1108	1106	1555	1541	*cis*-*p*-2-Menthen-1-ol	0.3
1117	1117	1374	1371	*allo*-Ocimene	0.1
1123	1124	n.d.	-	*p*-Menth-8-en-1-ol	0.2
1158	1158	1597	1598	Terpinene-4-ol	6.4
1169	1169	1692	1692	α-Terpineol	0.4
1177	1177	n.d.	*-*	*cis*-Piperitol	0.1
1187	1187	n.d.	*-*	*trans*-Piperitol	0.3
1214	1214	1591	1591	Thymyl methyl oxide	16.4
1223	1223	1601	1601	Carvacryl methyl oxide	0.1
1264	1264	1574	1574	Bornyl acetate	t
1479	1481	1726	1726	Bicyclogermacrene	0.3
1516	1516	2221	2215	Elemicine	t
1553	1551	2113	2213	Spathulenol	t
1696	1696	2350	*2350*	*E,E*-Farnesol	0.1
Monoterpene hydrocarbons					74.4
Oxygen containing monoterpenes					24.8
Sesquiterpene hydrocarbons					0.3
Oxygen containing sesquiterpenes					0.1
Other compounds					t
Total identified					99.6

Exp. RI^a^: Experimental retention indices on the SPB-1 column relative to C_8_–C_23_ *n*-alkanes. Ref. RI^a^: Reference retention indices in nonpolar column. Exp. RI^b^: Experimental retention on the SupelcoWax-10 column relative to C_8_ to C_23_ *n*-alkanes. Ref. RI^b^: Reference retention indices in polar column. * Compounds listed in order to their elution on the SPB-1 column.

**Table 3:** Antioxidant activity of aqueous extracts of *Crithmum maritimum* L. and BHA by DPPH and ABTS methods. The results are expressed as mean ± SD of three independent experiments (n = 3) performed in duplicate. The statistical analysis was performed with *p* < 0.05.

	IC_50_ (μg/mL) (DPPH)	IC_50_ (μg/mL) (ABTS)
Infusion	36.5 ± 1.4 *	37.3 ± 2.6 **
Decoction	44.7 ± 4.4 *	38.4 ± 1.8 **
BHA	4.5 ± 0.1 *	-

* Results are significants at *p* < 0.05, One-way ANOVA followed by Tukey’s test; ** Results are not significant at *p* < 0.05, paired *t*-test.

**Table 4:** Antibacterial activity of essential oil from *Crithmum maritimum* L. by the disc diffusion method. Results are expressed as mean ± SD.

Bacteria	Diameter of the Inhibition Halo (mm)
*Escherichia coli*	8.2 ± 1.2
*Staphylococcus aureus*	11.6 ± 1.5
*Bacillus cereus*	9.4 ± 1.2
*Lactobacillus plantarum*	18.2 ± 1.3

**Table 5:** MICs and MBCs results of *Crithmum maritimum* L. essential oil. All the determinations were performed in triplicate and the results expressed in mean *±* SD.

Bacteria	MIC (μL/mL)	MBC (μL/mL)
*Escherichia coli*	45.6 ± 0.1	91.3 ± 0.3
*Staphylococcus aureus*	22.8 ± 0.1	91.3 ± 0.3
*Bacillus cereus*	11.4 ± 0.1	w.e. *
*Lactobacillus plantarum*	11.4 ± 0.1	22.8 ± 0.1

* w.e.: without effect; lack of bactericidal effect.

## Data Availability

Not applicable.

## References

[1] Bozsik N., Cubillos J., Stalbek B., Vasa L., Magda R. (2022). Food security management in developing countries: Influence of economic factors on their food availability and access. PLoS ONE.

[2] Zenobi S., Fiorentini M., Ledda L., Deligios P., Aquilanti L., Orsini R. (2022). *Crithmum maritimum* L. Biomass Production in Mediterranean Environment. Agron.

[3] Ahmadi F., Mohammadkhani N., Servati M. (2022). Halophytes play important role in phytoremediation of salt-affected soils in the bed of Urmia Lake, Iran. Sci. Rep..

[4] Rodrigues M., Custódio L., Mecha D., Zengin G., Cziáky Z., Sotkó G., Pereira C. (2022). Nutritional and Phyto-Therapeutic Value of the Halophyte *Cladium mariscus* L. (Pohl.): A Special Focus on Seeds. Plants.

[5] Hawas U., El-Kassem L., Shaher F., Al-Farawati R., Ghandourah M. (2022). Phytochemical Compositions of Some Red Sea Halophyte Plants with Antioxidant and Anticancer Potentials. Molecules.

[6] Tan M., He F., Morris J., MacGregor G. (2022). Reducing daily salt intake in China by 1 g could prevent almost 9 million cardiovascular events by 2030: A modelling study. BMJ Nutr. Prev. Health.

[7] Kenao T., Jerôme C.S., Paraiso M., Belo M., Sopoh G., Agueh V. (2022). Dietary Sodium and Potassium Intakes and Salt Reduction Strategies: Systematic Review in Africa (2012–2022). Int. Arch. Public Health Community Med..

[8] Cury C., Banin V., Reis P., Caramori J., Barretti P., de Andrade L., Martin L. (2022). Association between urinary sodium excretion and hard outcomes in non-dialysis chronic kidney disease patients. BMC Nephrol..

[9] Shi H., Su X., Li C., Guo W., Wang L. (2022). Effect of a low-salt diet on chronic kidney disease outcomes: A systematic review and meta-analysis. BMJ Open.

[10] Renna M. (2018). Reviewing the prospects of sea fennel (*Crithmum maritimum* L.) as emerging vegetable crop. Plants.

[11] Zafeiropoulou V., Tomou E.-M., Ioannidou O., Karioti A., Skaltsa H. (2020). Sea fennel: Phytochemical analysis of Greek wild and cultivated *Crithmum maritimum* L. populations, based on HPLC-PDA-MS and NMR methods. J. Pharmacogn. Phytochem..

[12] Alves-Silva J., Guerra I., Gonçalves M., Cavaleiro C., Cruz M., Figueirinha A., Salgueiro L. (2020). Chemical composition of *Crithmum maritimum* L. essential oil and hydrodistillation residual water by GC-MS and HPLC-DAD-MS/MS, and their biological activities. Ind. Crops Prod..

[13] Petropoulos S., Karkanis A., Martins N., Ferreira I. (2018). Edible halophytes of the Mediterranean basin: Potential candidates for novel food products. Trends Food Sci. Technol..

[14] Piatti D., Angeloni S., Maggi F., Caprioli G., Ricciutelli M., Arnoldi L., Bosisio S., Mombelli G., Drenaggi E., Sagratini G. (2023). Comprehensive characterization of phytochemicals in edible sea fennel (*Crithmum maritimum* L., Apiaceae) grown in central Italy. J. Food Compos. Anal..

[15] Maoloni A., Cardinali F., Milanović V., Osimani A., Verdenelli M., Coman M., Aquilanti L. (2022). Exploratory Study for Probiotic Enrichment of a Sea Fennel (*Crithmum maritimum* L.) Preserve in Brine. Foods.

[16] Nabet N., Boudries H., Chougui N., Loupassaki S., Souagui S., Burló F., Hernández F., Carbonell-Barrachina Á., Madani K., Larbat R. (2017). Biological activities and secondary compound composition from *Crithmum maritimum* aerial parts. Int. J. Food Prop..

[17] Pedreiro S., da Ressurreição S., Lopes M., Cruz M., Batista T., Figueirinha A., Ramos F. (2021). *Crepis vesicaria* L. Subsp. taraxacifolia leaves: Nutritional profile, phenolic composition and biological properties. Int. J. Environ. Res. Public Health.

[18] Figueirinha A., Cruz M., Francisco V., Lopes M., Batista M. (2010). Anti-Inflammatory Activity of *Cymbopogon citratus* Leaf Infusion in Lipopolysaccharide-Stimulated Dendritic Cells: Contribution of the Polyphenols. J. Med. Food..

[19] Giordano A., Morales-Tapia P., Moncada-Basualto M., Pozo-Martínez J., Olea-Azar C., Nesic A., Cabrera-Barjas G. (2022). Polyphenolic Composition and Antioxidant Activity (ORAC, EPR and Cellular) of Different Extracts of Argylia radiata Vitroplants and Natural Roots. Molecules.

[20] Couto J., Figueirinha A., Batista M., Paranhos A., Nunes C., Gonçalves L., Marto J., Fitas M., Pinto P., Ribeiro H. (2020). *Fragaria vesca* L. Extract: A promising cosmetic ingredient with antioxidant properties. Antioxidants.

[21] Rosca A., Castro J., Sousa L., França A., Cavaleiro C., Salgueiro L., Cerca N. (2022). Six Bacterial Vaginosis-Associated Species Can Form an In Vitro and Ex Vivo Polymicrobial Biofilm that Is Susceptible to Thymbra capitata Essential Oil. Front. Cell. Infect. Microbiol..

[22] Cardoso O., Donato M., Luxo C., Almeida N., Liberal J., Figueirinha A., Batista M. (2018). Anti-*Helicobacter pylori* potential of *Agrimonia eupatoria* L. and *Fragaria vesca*. J. Funct. Foods.

[23] Liberal J., Francisco V., Costa G., Figueirinha A., Amaral M., Marques C., Girão H., Lopes M., Cruz M., Batista M. (2014). Bioactivity of Fragaria vesca leaves through inflammation, proteasome and autophagy modulation. J. Ethnopharmacol..

[24] Francisco V., Figueirinha A., Costa G., Liberal J., Lopes M., García-Rodríguez C., Geraldes C., Cruz M., Batista M. (2014). Chemical characterization and anti-inflammatory activity of luteolin glycosides isolated from lemongrass. J. Funct. Foods.

[25] Matos P., Figueirinha A., Ferreira I., Cruz M., Batista M. (2019). *Acanthus mollis* L. leaves as source of anti-inflammatory and antioxidant phytoconstituents. Nat. Prod. Res..

[26] Borges P., Pedreiro S., Baptista S., Geraldes C., Batista M., Silva M., Figueirinha A. (2021). Inhibition of α-glucosidase by flavonoids of Cymbopogon citratus (DC) Stapf. J. Ethnopharmacol..

[27] Alotaibi B., Ijaz M., Buabeid M., Kharaba Z., Yaseen H., Murtaza G. (2021). Therapeutic Effects and Safe Uses of Plant-Derived Polyphenolic Compounds in Cardiovascular Diseases: A Review. Drug Des. Devel. Ther..

[28] Gasmi A., Mujawdiya P., Noor S., Lysiuk R., Darmohray R., Piscopo S., Lenchyk L., Antonyak H., Dehtiarova K., Shanaida M. (2022). Polyphenols in Metabolic Diseases. Molecules.

[29] Pereira C., Barreira L., da Rosa Neng N., Nogueira J., Marques C., Santos T., Varela J., Custódio L. (2017). Searching for new sources of innovative products for the food industry within halophyte aromatic plants: In vitro antioxidant activity and phenolic and mineral contents of infusions and decoctions of *Crithmum maritimum* L. Food Chem. Toxicol..

[30] Souid A., Della Croce C., Frassinetti S., Gabriele M., Pozzo L., Ciardi M., Abdelly C., Hamed K.B., Magné C., Longo V. (2021). Nutraceutical Potential of Leaf Hydro-Ethanolic Extract of the Edible Halophyte *Crithmum maritimum* L. Molecules.

[31] Anand S., Sati N. (2013). Artificial Preservatives and Their Harmful Effects: Looking Toward Nature for Safer Alternatives. Int. J. Pharm. Sci. Res. IJPSR.

[32] Chinyere S. (2021). Analysis of Health Consequences of Preservatives on Agricultural Foods. Off. Publ. Direct Res. J. Agric. Food Sci..

[33] Halla N., Fernandes I., Heleno S., Costa P., Boucherit-Otmani Z., Boucherit K., Rodrigues A., Ferreira I., Barreiro M. (2018). Cosmetics Preservation: A Review on Present Strategies. Mol. A J. Synth. Chem. Nat. Prod. Chem..

[34] Yim E., Nole K., Tosti A. (2014). Contact dermatitis caused by preservatives. Dermatitis.

[35] Attebäck M., Hedin B., Mattsson S. (2022). Formulation Optimization of Extemporaneous Oral Liquids Containing Naloxone and Propranolol for Pediatric Use. Sci. Pharm..

[36] Galié S., García-Gutiérrez C., Miguélez E., Villar C., Lombó F. (2018). Biofilms in the food industry: Health aspects and control methods. Front. Microbiol..

[37] Lorenzo J., Munekata P., Dominguez R., Pateiro M., Saraiva J., Franco D. (2018). Main Groups of Microorganisms of Relevance for Food Safety and Stability: General Aspects and Overall Description. Innov. Technol. Food Preserv..

[38] Bashir A., Lambert P. (2020). Microbiological study of used cosmetic products: Highlighting possible impact on consumer health. J. Appl. Microbiol..

[39] Union P. (2009). Regulation (EC) No 1223/2009 of the European Parliament and of the Council of 30 November 2009 on cosmetic products (recast) Text with EEA relevance. Off. J. Eur. Union.

[40] Scientific Committee on Consumer Products (SCCP) (2018). The SCCP’s Notes of Guidance for the Testing of Cosmetic Ingredients and Their Safety Evaluation.

[41] Horwitz W., AOAC International (2000). Official Methods of Analysis of AOAC International.

[42] Adams R. (2007). Identification of Essential Oil Components by Gas Chromatography/Mass Spectrometry.

[43] Linstrom P., Mallard W. (2019). NIST Chemistry WebBook, NIST Stand. Ref. Database Number 69. Natl. Inst. Stand. Technol..

[44] McLafferty F. (2009). Wiley Registry of Mass Spectral Data 9th/NIST 08.

[45] Re R., Pellegrini N., Proteggente A., Pannala A., Yang M., Rice-Evans C. (1999). Antioxidant activity applying an improved ABTS radical cation decolorization assay. Free Radic. Biol. Med..

[46] Carvalho M., Albano H., Teixeira P. (2018). In Vitro Antimicrobial Activities of Various Essential Oils Against Pathogenic and Spoilage Microorganisms. J. Food Qual. Hazards Control.

[47] Balouiri M., Sadiki M., Ibnsouda S. (2016). Methods for in vitro evaluating antimicrobial activity: A review. J. Pharm. Anal..

[48] Lopes M., Roque M., Cavaleiro C., Ramos F. (2021). Nutrient value of Salicornia ramosissima—A green extraction process for mineral analysis. J. Food Compos. Anal..

[49] Martins-Noguerol R., Matías L., Pérez-Ramos I., Moreira X., Muñoz-Vallés S., Mancilla-Leytón J., Francisco M., García-González A., DeAndrés-Gil C., Martínez-Force E. (2022). Differences in nutrient composition of sea fennel (*Crithmum maritimum*) grown in different habitats and optimally controlled growing conditions. J. Food Compos. Anal..

[50] Karkanis A., Polyzos N., Kompocholi M., Petropoulos S. (2022). Rock Samphire, a Candidate Crop for Saline Agriculture: Cropping Practices, Chemical Composition and Health Effects. Appl. Sci..

[51] Da Ressurreição S., Pedreiro S., Batista M., Figueirinha A. (2022). Effect of Phenolic Compounds from Cymbopogon citratus (DC) Stapf. Leaves on Micellar Solubility of Cholesterol. Molecules.

[52] Mekinić I., Blažević I., Mudnić I., Burčul F., Grga M., Skroza D., Jerčić I., Ljubenkov I., Boban M., Miloš M. (2016). Sea fennel (*Crithmum maritimum* L.): Phytochemical profile, antioxidative, cholinesterase inhibitory and vasodilatory activity. J. Food Sci. Technol..

[53] Alemán A., Marín D., Taladrid D., Montero P., Gómez-Guillén M.C. (2019). Encapsulation of antioxidant sea fennel (*Crithmum maritimum*) aqueous and ethanolic extracts in freeze-dried soy phosphatidylcholine liposomes. Food Res. Int..

[54] Rojas-González A., Figueroa-Hernández C., González-Rios O., Suárez-Quiroz M., González-Amaro R., Hernández-Estrada Z., Rayas-Duarte P. (2022). Coffee Chlorogenic Acids Incorporation for Bioactivity Enhancement of Foods: A Review. Molecules.

[55] Vergara-Salinas J., Pérez-Jiménez J., Torres J., Agosin E., Pérez-Correa J. (2012). Effects of temperature and time on polyphenolic content and antioxidant activity in the pressurized hot water extraction of deodorized thyme (*Thymus vulgaris*). J. Agric. Food Chem..

[56] Kaczorová D., Karalija E., Dahija S., Bešta-Gajević R., Parić A., Zeljković S.Ć. (2021). Influence of Extraction Solvent on the Phenolic Profile and Bioactivity of Two Achillea Species. Molecules.

[57] Özcan M., Akgül A., Başcr K., Özck T., Tabanca N. (2001). Essential oil composition of sea fennel (*Crithmum maritimum*) form Turkey. Nahr./Food.

[58] Kulisic-Bilusic T., Blažević I., Dejanović B., Miloš M., Pifat G. (2010). Evaluation of the Antioxidant Activity of Essential Oils from Caper (*Capparis spinosa*) and Sea Fennel (*Crithmum Maritimum*) by Different Methods. J. Food Biochem..

[59] Ruberto G., Baratta M., Deans S., Dorman H. (2000). Antioxidant and antimicrobial activity of Foeniculum vulgare and *Crithmum maritimum* essential oils. Planta Med..

[60] Senatore F., Napolitano F., Ozcan M. (2000). Composition and antibacterial activity of the essential oil from *Crithmum maritimum* L. (Apiaceae) growing wild in Turkey. Flavour Fragr. J..

[61] Şanli A., Karadoğan T. (2017). Geographical Impact on Essential Oil Composition of Endemic Kundmannia Anatolica Hub.-Mor. (Apiaceae). African J. Tradit. Complement. Altern. Med..

[62] Siracusa L., Kulisic-Bilusic T., Politeo O., Krause I., Dejanovic B., Ruberto G. (2011). Phenolic composition and antioxidant activity of aqueous infusions from *Capparis spinosa* L. and *Crithmum maritimum* L. before and after submission to a two-step in vitro digestion model. J. Agric. Food Chem..

[63] Durmaz L., Kiziltas H., Guven L., Karagecili H., Alwasel S., Gulcin İ. (2022). Antioxidant, Antidiabetic, Anticholinergic, and Antiglaucoma Effects of Magnofluorine. Molecules.

[64] Weber R. (2014). Adverse Reactions to the Antioxidants Butylated Hydroxyanisole and Butylated Hydroxytoluene. Food Allergy: Adverse Reactions to Foods and Food Additives.

[65] Caleja C., Barros L., Antonio A., Beatriz M., Oliveira P., Ferreira I. (2017). A comparative study between natural and synthetic antioxidants: Evaluation of their performance after incorporation into biscuits. Food Chem..

[66] Chen J., Yang J., Ma L., Li J., Shahzad N., Kim C. (2020). Structure-antioxidant activity relationship of methoxy, phenolic hydroxyl, and carboxylic acid groups of phenolic acids. Sci. Rep..

[67] Wang L., Pan X., Jiang L., Chu Y., Gao S., Jiang X., Zhang Y., Chen Y., Luo S., Peng C. (2022). The Biological Activity Mechanism of Chlorogenic Acid and Its Applications in Food Industry: A Review. Front. Nutr..

[68] Pedreiro S., Figueirinha A., Silva A., Ramos F. (2021). Bioactive Edible Films and Coatings Based in Gums and Starch: Phenolic Enrichment and Foods Application. Coatings.

[69] Xue N., Liu Y., Jin J., Ji M., Chen X. (2022). Chlorogenic Acid Prevents UVA-Induced Skin Photoaging through Regulating Collagen Metabolism and Apoptosis in Human Dermal Fibroblasts. Int. J. Mol. Sci..

[70] Neelakandan M., Manoharan S., Muralinaidu R., Thara J. (2022). Tumor preventive and antioxidant efficacy of chlorogenic acid–loaded chitosan nanoparticles in experimental skin carcinogenesis. Naunyn. Schmiedebergs. Arch. Pharmacol..

[71] Lou Z., Wang H., Zhu S., Ma C., Wang Z. (2011). Antibacterial activity and mechanism of action of chlorogenic acid. J. Food Sci..

[72] Su M., Liu F., Luo Z., Wu H., Zhang X., Wang D., Zhu Y., Sun Z., Xu W., Miao Y. (2019). The Antibacterial Activity and Mechanism of Chlorogenic Acid Against Foodborne Pathogen *Pseudomonas aeruginosa*. Foodborne Pathog. Dis..

[73] Sun Z., Zhang X., Wu H., Wang H., Bian H., Zhu Y., Xu W., Liu F., Wang D., Fu L. (2020). Antibacterial activity and action mode of chlorogenic acid against Salmonella Enteritidis, a foodborne pathogen in chilled fresh chicken. World J. Microbiol. Biotechnol..

[74] Martínez G., Regente M., Jacobi S., Del Rio M., Pinedo M., de la Canal L. (2017). Chlorogenic acid is a fungicide active against phytopathogenic fungi. Pestic. Biochem. Physiol..

[75] Pitt T., McClure J., Parker M., Amézquita A., McClure P. (2015). Bacillus cereus in personal care products: Risk to consumers. Int. J. Cosmet. Sci..

[76] Tewari A., Abdullah S. (2015). Bacillus cereus food poisoning: International and Indian perspective. J. Food Sci. Technol..

[77] Kadariya J., Smith T., Thapaliya D. (2014). Staphylococcus aureus and Staphylococcal Food-Borne Disease: An Ongoing Challenge in Public Health. Biomed Res. Int..

[78] Kim H., Seok Y., Cho T., Rhee M. (2020). Risk factors influencing contamination of customized cosmetics made on-the-spot: Evidence from the national pilot project for public health. Sci. Rep..

[79] Muhammad Z., Ramzan R., Abdelazez A., Amjad A., Afzaal M., Zhang S., Pan S. (2019). Assessment of the Antimicrobial Potentiality and Functionality of *Lactobacillus plantarum* Strains Isolated from the Conventional Inner Mongolian Fermented Cheese Against Foodborne Pathogens. Pathogens.

[80] Jo C., Myung C., Yoon Y., Ahn B., Min J., Seo W., Lee D., Kang H., Heo Y., Choi H. (2022). The Effect of Lactobacillus plantarum Extracellular Vesicles from Korean Women in Their 20s on Skin Aging. Curr. Issues Mol. Biol..

[81] Sánchez-Hernández E., Buzón-Durán L., Andrés-Juan C., Lorenzo-Vidal B., Martín-Gil J., Martín-Ramos P. (2021). Physicochemical characterization of *Crithmum maritimum* L. and daucus carota subsp. gummifer (syme) hook.fil. and their antimicrobial activity against apple tree and grapevine phytopathogens. Agronomy.

[82] D’agostino G., Giambra B., Palla F., Bruno M., Badalamenti N. (2021). The Application of the Essential Oils of Thymus vulgaris L. and *Crithmum maritimum* L. as Biocidal on Two Tholu Bommalu Indian Leather Puppets. Plants.

[83] Li L., Li Z.-W., Yin Z.-Q., Wei Q., Jia R.-Y., Zhou L.-J., Xu J., Song X., Zhou Y., Du Y.-H. (2014). Antibacterial activity of leaf essential oil and its constituents from *Cinnamomum longepaniculatum*. Int. J. Clin. Exp. Med..

[84] Guimarães A., Meireles L., Lemos M., Guimarães M., Endringer D., Fronza M., Scherer R. (2019). Antibacterial Activity of Terpenes and Terpenoids Present in Essential Oils. Molecules.

[85] Mahato D., Lee K., Kamle M., Devi S., Dewangan K., Kumar P., Kang S. (2019). Aflatoxins in Food and Feed: An Overview on Prevalence, Detection and Control Strategies. Front. Microbiol..

[86] Rychen G., Aquilina G., Azimonti G., Bampidis V., de Lourdes Bastos M., Bories G., Cocconcelli P., Flachowsky G., Gropp J., Kolar B. (2018). Safety and efficacy of cumin tincture (*Cuminum cyminum* L.) when used as a sensory additive for all animal species. EFSA J..

[87] EFSA Panel on Additives and Products or Substances used in Animal Feed (FEEDAP) (2016). Safety and efficacy of BIOSTRONG^®^ 510 (essential oil of thyme and star anise) for chickens and minor avian species for fattening and rearing to point of lay. EFSA J..

[88] Jha R., Fouhse J., Tiwari U., Li L., Willing B. (2019). Dietary fiber and intestinal health of monogastric animals. Front. Vet. Sci..

[89] Sharmeen J., Mahomoodally F., Zengin G., Maggi F., Montesano D., Petrelli R. (2021). Essential Oils as Natural Sources of Fragrance Compounds for Cosmetics and Cosmeceuticals. Molecules.

[90] Guzmán E., Lucia A. (2021). Essential Oils and Their Individual Components in Cosmetic Products. Cosmetics.

[91] Ullah H., De Filippis A., Baldi A., Dacrema M., Esposito C., Garzarella E.U., Santarcangelo C., Tantipongpiradet A., Daglia M. (2021). Beneficial Effects of Plant Extracts and Bioactive Food Components in Childhood Supplementation. Nutrients.

